# New Program and Directions at the National Institute of Standards and Technology

**DOI:** 10.6028/jres.095.002

**Published:** 1990

**Authors:** Donald R. Johnson

**Affiliations:** National Institute of Standards and Technology, Gaithersburg, MD 20899

**Keywords:** Advanced Technology Program, Clearinghouse for State Technology Programs, Manufacturing Technology Centers, State Technology Extension Program, Technology Services

## Abstract

The Trade Act of 1988 created the National Institute of Standards and Technology (NIST) from the National Bureau of Standards (NBS). In addition to explicitly defining and reconfirming the traditional measurement services, the law assigned new responsibilities to NIST to assist U.S. industry in capitalizing on new technologies developed in the U.S. scientific and technical community at a faster rate. This article decribes the new programs being established at NIST to comply with this mandate and the new organizational unit at NIST that brings together the traditional services and these new programs.

## 1. Introduction

The rapid loss of competitiveness of American industry in international markets is an extremely serious problem with wide-ranging consequences for the United States’ material well-being, security and political influence. Its causes are many, but among them certainly are the slow rate at which new technology is incorporated in commercial products and processes, and the lack of attention paid to manufacturing. There is a clear need to compete in world markets with high-value-added products, incorporating the latest innovations, manufactured in short runs with flexible manufacturing methods. Research, management, and manufacturing methods that support change and innovation are key ingredients needed to enhance our Nation’s competitive position.

Many ideas originating in the American scientific and technical community are being commercially exploited in other parts of the world. As a nation, we have been slow to capitalize on new technology developed from America’s own intellectual capability. In the past, small and mid-sized companies have led U.S. industry in innovation. Our government must now find ways to help such companies meet the demands of global competition, when the speed with which firms are able to translate innovations into quality commercial products and processes is of utmost importance.

The Omnibus Trade and Competitiveness Act signed into law on August 23, 1988 is the result of policymakers’ endeavors to create a new framework and environment that can enhance the rapid commercialization of technology. Among other things the Act created the National Institute of Standards and Technology (NIST) from the National Bureau of Standards (NBS) and assigned to it several new and expanded functions which build on the technical expertise of NBS. NIST will maintain the traditional functions of NBS in support of U.S. industry and will continue to offer the full array of measurement and quality assurance services including calibration services, standard reference materials, standard reference data, and measurement assurance programs.

The NIST resources are still quite modest when compared to research and development expenditures by industry and the federal government, or with the more than $550 million expenditure by the various states for technology development and commercialization. However, the new programs being developed by NIST in response to the new legislative assignments are designed to be collaborative, highly leveraged, and serve as examples to be followed by others with greater resources.

## 2. New NIST Organizational Unit

**Figure 1:**
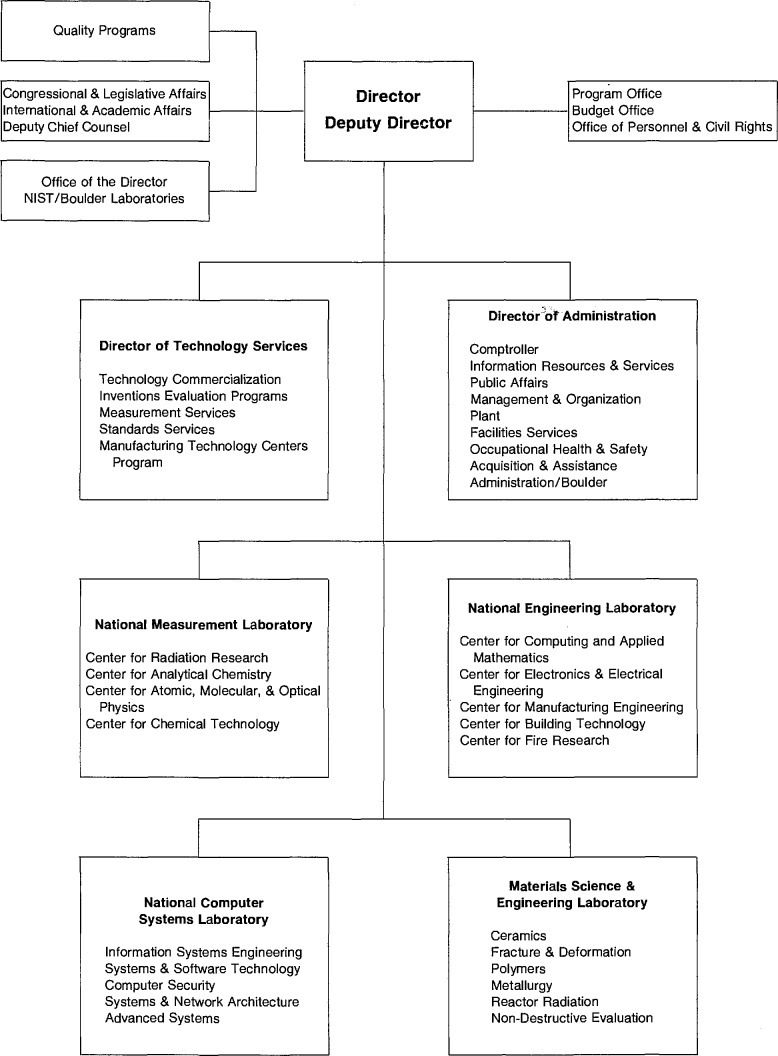
Organizational placement of Technology Services within NIST.

**Figure 2:**
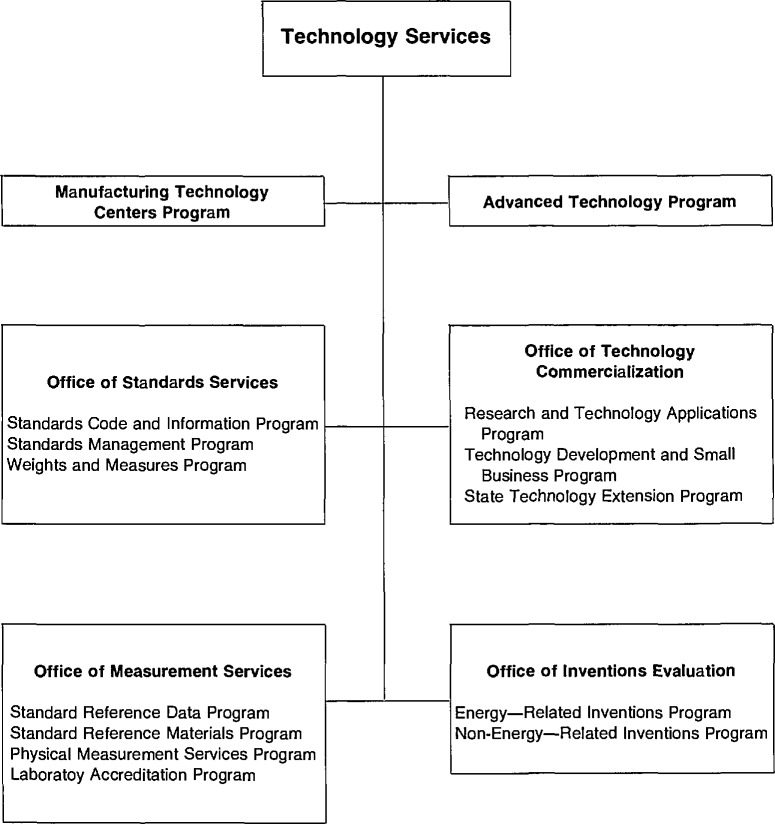
Organizational Structure of Technology Services.

A new major organizational unit, Technology Services (TS), was established in NIST to bring together the new assignments, which are mainly extramural, and the traditional NIST (NBS) services which have been, and are now more than ever, an important link in our Nation’s efforts to improve its industrial competitiveness. Figure 1 shows the organizational placement of the new unit within NIST. Figure 2 shows the organizational structure of Technology Services in more detail.

## 3. New Programs

The Act assigns to NIST four new major programs designed to assist private sector initiatives capitalize on technological innovations; advance R&D projects which can be optimized for commercial and industrial applications; and promote shared risks, accelerated development, and pooling of skills necessary to strengthen America’s manufacturing skills. The new programs are:

- the Regional Centers for the Transfer of Manufacturing Technology,
- the State Technology Extension Program,
- the Advanced Technology Program,
- the Clearinghouse for State Technology Programs.

### 3.1 Regional Centers for the Transfer of Manufacturing Technology

The objective of the regional centers is to bring modern automated manufacturing technology to small and mid-sized manufacturing firms. The program focuses on technologies appropriate to firms in a selected geographic region and emphasizes “hands on” experience and “off the shelf” technologies. Three organizations have been selected to become the first NIST Regional Manufacturing Technology Centers: The Great Lakes Manufacturing Technology Center at the Cleveland Advanced Manufacturing Program in Cleveland, Ohio; The Northeast Manufacturing Technology Center at Rensselaer Polytechnic Institute in Troy, New York; and The Southeast Manufacturing Technology Center at the University of South Carolina in Columbia, South Carolina. NIST has now established cooperative working agreements with each of these organizations.

### 3.2 State Technology Extension Program

The objective of the extension services program is to improve the use of technology, particularly federal technology, by small and mid-sized businesses. A number of federal, state, and local sponsored extension services already exist, but most of the services offered are focused on business assistance rather than technology. In addition, most of the organizations providing extension services do not have sufficient technically trained personnel nor the technical resources needed to help small companies with their technological needs. This NIST program will help to coordinate the state and local extension services with federal technology transfer programs by working with the existing delivery network; provide technology assistance to extension services as appropriate; develop and conduct workshops/seminars on technological issues; and expand the distribution and utilization of NIST services.

### 3.3 Advanced Technology Program (ATP)

The objective of this program is to accelerate the commercialization of scientific discoveries and manufacturing technologies, particularly by small entrepreneurial firms. This program will provide limited federal funding to encourage and leverage private sources of support for developing generic technology, developing new products from specific projects, and improving existing manufacturing processes.

NIST has developed a preliminary ATP program plan that calls for the initiation of eight operating program components (see table 1) that together would:

- encourage U.S. business to look to the future and to improve their competitive positions through technological innovation,
- capture greater civilian market potential from existing Federal investment on basic research,
- systematically vector different aspects of Federal research investments to follow-on funding from state and local governments.

### 3.4 Clearinghouse for State Technology Programs

The objective of this program is to develop a central base of information on programs already in place and document the results that can be measured as a resource for state and local governments to utilize when deciding on new technology investments. NIST will acquire information through the development of a network of technical contacts within the state and local policy level staff, and through the collection of information on current programs. The clearinghouse will provide the type of information needed at the state and local level by governors, county executives, mayors, and other decision makers as they plan new programs and make policy decisions. The total investment at the state level is large and the influence on the overall direction of U.S. high technology and, hence, the impact on the nation’s balance of trade will be substantial. The availability of a quality data base on state technology programs is an essential resource for the decision makers.

## 4. Conclusion

Our new name, National Institute of Standards and Technology, reflects the broadened role and the new responsibilities. NIST will continue to serve as the Nation’s central laboratory for developing and disseminating measurement standards and scientific data for service, engineering, manufacturing, commerce, industry, and education. The combination of our new assignments and the traditional NBS measurement and quality assurance services under the new organizational unit (TS) will enable NIST to focus more effectively on its new purpose, “to assist industry in the development of technology and procedures needed to improve quality, to modernize manufacturing processes, to ensure product reliability, manufacturability, functionality, and cost-effectiveness, and to facilitate the more rapid commercialization… of products based on new scientific discoveries.”

## Figures and Tables

**Table 1:** Advanced Technology Program (ATP) options

**•Emerging Technologies Consortia**	**•SBIR Phase III Technology Development**
Provide start-up support for joint R&D ventures to rapidly develop generic technologies having exceptional long-term commerical promise.	Provide follow-on support for commercially promising Small Business Innovation Research (SBIR) Phase II Projects
**•Manufacturing Research Consortia**	**•Invention Evaluation**
Provide start-up support for joint R&D ventures to create generic improvements in manufacturing technology or improved productivity and quality control	Provide seed support for highly ranked, commercially promising inventions from extension services program
**•Business-Federal Laboratory Partnership**	**•Business-State Partnership**
Provide funding matched with private sector funds to extract commercially promising technology from federal labs	Provide leverage funding with state programs to insure local follow-on support for commerically promising projects
**•Business-University Partnership**	**•Prototype Engineering Research**
Provide funding matched with private sector funds to extract commercially promising technologies from universities	Provide support for successfully completed ATP projects with exceptional commerical potential but low private sector funding appeal

